# Optical Coherence Tomography

**DOI:** 10.1100/tsw.2007.29

**Published:** 2007-01-26

**Authors:** Pier Alberto Testoni

**Affiliations:** Division of Gastroenterology, Vita-Salute San Raffaele University, Scientific Institute San Raffaele, Milan, Italy

**Keywords:** optical coherence tomography, Barrett's epithelium, dysplasia, adenocarcinoma, gastrointestinal tract, pancreatico-biliary ductal system

## Abstract

Optical coherence tomography (OCT) is an optical imaging modality that performs high-resolution, cross-sectional, subsurface tomographic imaging of the microstructure of tissues. The physical principle of OCT is similar to that of B-mode ultrasound imaging, except that it uses infrared light waves rather than acoustic waves. The *in vivo* resolution is 10–25 times better (about 10 µm) than with high-frequency ultrasound imaging, but the depth of penetration is limited to 1–3 mm, depending on tissue structure, depth of focus of the probe used, and pressure applied to the tissue surface. In the last decade, OCT technology has evolved from an experimental laboratory tool to a new diagnostic imaging modality with a wide spectrum of clinical applications in medical practice, including the gastrointestinal tract and pancreatico-biliary ductal system. OCT imaging from the gastrointestinal tract can be done in humans by using narrow-diameter, catheter-based probes that can be inserted through the accessory channel of either a conventional front-view endoscope, for investigating the epithelial structure of the gastrointestinal tract, or a side-view endoscope, inside a standard transparent ERCP (endoscopic retrograde cholangiopancreatography) catheter, for investigating the pancreatico-biliary ductal system. The esophagus and esophagogastric junction have been the most widely investigated organs so far; more recently, duodenum, colon, and the pancreatico-biliary ductal system have also been extensively investigated. OCT imaging of the gastrointestinal wall structure is characterized by a multiple-layer architecture that permits an accurate evaluation of the mucosa, lamina propria, muscularis mucosae, and part of the submucosa. The technique may therefore be used to identify preneoplastic conditions of the gastrointestinal tract, such as Barrett's epithelium and dysplasia, and evaluate the depth of penetration of early-stage neoplastic lesions. OCT imaging of the pancreatic and biliary ductal system could improve the diagnostic accuracy for ductal epithelial changes, and the differential diagnosis between neoplastic and non-neoplastic lesions.

Optical coherence tomography (OCT) is an optical imaging modality introduced in 1991[1] that performs high-resolution, cross-sectional, subsurface tomographic imaging of the microstructure in materials and biologic systems by measuring back-scattered or back-reflected infrared light.

The physical principle of OCT is similar to that of B-mode ultrasound imaging, except that the intensity of infrared light, rather than sound waves, is measured. Wavelengths of the infrared light used in OCT are one to two orders of magnitude higher than ultrasound wavelengths, so OCT technology can yield a lateral and axial spatial resolution of about 10 μm, which is 10- to 25-fold better than that of available high-frequency ultrasound imaging. The spatial resolution of OCT images is nearly equivalent to that of histologic sections. The depth of penetration of OCT imaging is approximately 1–3 mm, depending on tissue structure, depth of focus of the probe used, and pressure applied to the tissue surface. Although the progressive increase in ultrasound resolution is accompanied by a corresponding decease in depth of penetration, a similar trade-off between resolution and depth of penetration does not occur in OCT imaging.

In the last decade, OCT technology has evolved from an experimental laboratory tool to a new diagnostic imaging modality with a wide spectrum of clinical applications in medical practice, including the gastrointestinal tract and pancreatico-biliary ductal system.

## PRINCIPLES OF OPERATION OF OCT

OCT devices use a low-power, infrared light with a wavelength ranging from 750–1300 nm in which the only limiting factor is the scattering of light. Scattering occurs when the light interacts with the tissue surface and the image formation depends on the difference in optical back-scattering properties of the tissue.

OCT images are generated from measuring the echo time delay and the intensity of back-scattered light. Because the velocity of light is extremely high, optical echoes cannot be measured by direct electronic detection, but by means of a low-coherence interferometry that measures the interference of two incident light beams that are derived from a single source of low-coherence light. Low-coherence light can be generated by compact superluminescent semiconductor diodes or other sources, such as solid-state lasers.

Using low-coherence interferometry, the light reflected or back-scattered from inside the specimen is measured by correlating with light that has traveled a known reference path. One interferometer arm contains a modular probe that focuses and scans the light onto the tissue sample, also collecting the back-scattered light; the second interferometer arm is a reference path with a translating mirror or scanning delay line[2].

Lights reflected from the specimen and reference beam are combined at a detector, and the interference between the two beams is measured. Optical interference between the light from the sample and reference path occurs only when the distance traveled by the light in both paths matches to within the coherence length of the light[3].

The coherence length of the light source, which is inversely proportional to its spectral bandwidth, determines the axial or depth resolution of OCT; the shorter the coherence length of the light source, the better is the in-depth spatial resolution. The spatial width of the scanning beam determines the lateral or transverse resolution. The image penetration depth is determined by the absorption and scattering properties of the sample surface.

In OCT, two-dimensional, cross-sectional images of tissue microstructure are constructed by scanning the optical beam and performing multiple axial measurements of back-scattered light at different transverse positions. The resulting dataset is a two-dimensional array that represents the displayed image as a grey-scale or false-color image.

Three types of scanning patterns are available for OCT imaging: radial[4,5,6], longitudinal[7,8], and transverse[9]. The radial-scan probe directs the OCT beam radially, giving images that are displayed in a "radar-like", circular plot. Radial scanning can easily image large areas of tissue by moving the probe forth and back over the tissue surface, and has the highest definition when the probe is inserted within a small-diameter lumen, because the OCT images become progressively coarser when a large-diameter lumen is scanned, due to the progressive increase of pixel spacing with increasing the distance between the probe and the tissue. The linear and transverse probes scan the longitudinal and transverse positions of the OCT beam at a fixed angle, generating rectangular images of longitudinal and transverse planes at a given angle with respect to the probe. Linear scanning has the advantage that pixel spacing in the transverse direction is uniform and can better image a definite area of the scanned tissue, especially in the presence of largediameter and noncircular lumens, where maintaining constant distance from the probe to the surface over the entire circumferential scan may be impossible. Transverse scanning modality provides a better depth of field. Depth of field is the range of distances from the probe over which optimal resolution of scanning can be obtained; current OCT scans permit imaging depths of up to 2–3 mm in tissues, by using probes with different focuses.

## OCT TECHNIQUE FOR THE GASTROINTESTINAL TRACT AND PANCREATICO-BILIARY DUCTAL SYSTEM

OCT imaging from the gastrointestinal (GI) tract can be done in humans by using narrow-diameter, catheter-based probes[10]. The probe is detachable from the OCT main unit, making it reprocessable between procedures.

The probe can be inserted through the accessory channel of either a conventional front-view endoscope, for investigating the epithelial structure of the GI tract, or a side-view endoscope, inside a standard transparent ERCP (endoscopic retrograde cholangiopancreatography) catheter, for investigating the pancreatico-biliary ductal system.

In our studies[7], a near-focus OCT probe (Pentax, Lightlab Imaging, Westford, MA) was used, with a penetration depth of about 1 mm and a resolution of approximately 10 μm. The probe operates at 1.2- to 1.4-μm center wavelength (nominal value: 1.3 μm), with a scan frequency ranging from 1000–4000 kHz (nominal value: 3125 kHz). Radial and longitudinal scanning resolutions have an operating range in tissue of 15–20 μ (nominal value: 18 μ) and 21–27 μ (nominal value: 24 μ), respectively. Infrared light is delivered to the imaging site through a single optical fiber 0.006" diameter. The OCT probe is assembled in a catheter with an outer diameter of 1.2 mm, The catheter-based probe consists of a rotating probe encased in a transparent outer sheath, which remains stationary while the rotating probe has a pull-back movement of 1 mm/sec, with an acquisition rate of 10 frames per second. By this technique, a segment of tissue 5.5 cm long can be filmed over a 55-sec period.

OCT scanning can be done by maintaining the probe placed lightly or firmly on the wall of the GI tract. When the probe is placed lightly on the mucosal surface, the depth of penetration is limited mainly to the superficial submucosa; by this way, the superficial epithelium, lamina propria, and the upper part of the submucosa are clearly visualized. When the probe is placed firmly against the mucosal surface, the submucosa and muscularis propria can be clearly visualized, but details of the superficial layers of the mucosa are lost. When the OCT probe is held in strict contact with the tissue surface, as occurs when it is inserted across strictures of the pancreatico-biliary ductal system, the superficial epithelium may appear compressed and difficult to evaluate.

## CLINICAL APPLICATION OF OCT IN THE GASTROINTESTINAL TRACT

Several *in vitro* studies demonstrated the feasibility of OCT in the GI tract. In these studies, the GI tract wall was identified as a multiple-layer structure characterized by a sequence of hyper- and hyporeflective layers, with a variable homogenicity of the back-scattered signal[11,12]. Neoplastic and normal tissue also showed different light back-scattering patterns[13]. However, the optical properties of nonliving tissues are different from tissue *in vivo*.

Subsequent studies were therefore performed in *ex vivo* tissue specimens and aimed at comparing OCT imaging with histology to assess the reliability of the OCT technique to identify and recognize the GI wall structure. OCT was shown to clearly differentiate the layer structure of the GI wall. In the esophagus, the epithelium appeared clearly recognizable and distinguishable from the lamina propria and muscularis mucosa. In the stomach and colon, the mucosa appeared distinguishable from the submucosa[14].

## Esophagus

The esophagus and the esophagogastric junction have been the most widely investigated organs so far. OCT imaging characteristics of normal esophageal wall structure were identified by comparing the *ex vivo* measurements of layer thickness and histology in the same specimen of a normal esophagus[7]. Using this method, the authors were able to define which OCT-imaged layers corresponded to each esophageal wall component. *In vivo* studies were also able to recognize the multiple-layer structure of the esophagus[9,15,16]. The esophagogastric junction appears to be clearly recognizable at OCT investigation because the stomach wall shows a different OCT pattern, characterized by the presence of a vertical crypt-and-pit architecture of the mucosa that changes abruptly to the horizontal, layered tissue architecture of the esophageal squamous epithelium.

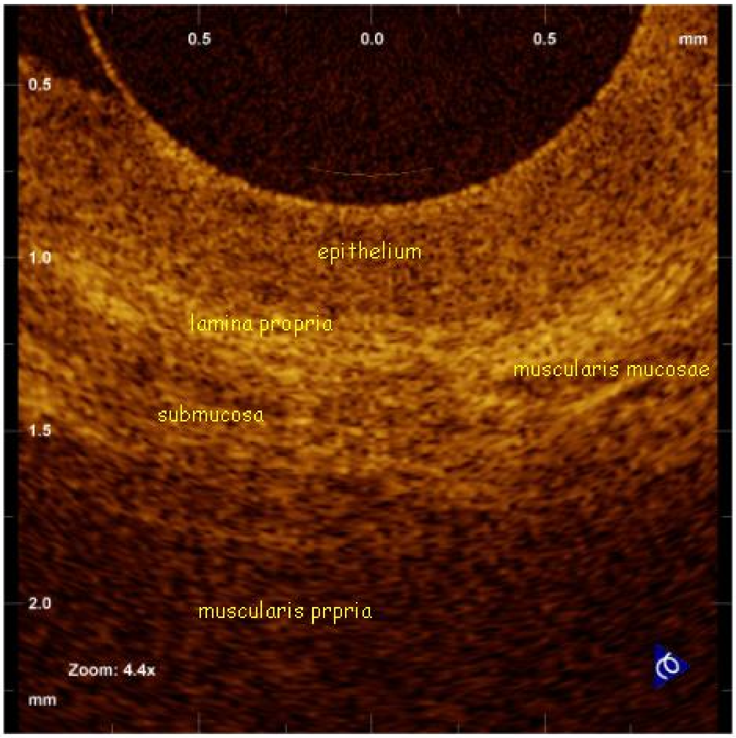

**FIGURE 1.** Magnification of OCT image showing normal esophageal wall. The OCT image shows a multiple-layer structure characterized by a superficial, weakly scattering (hyporeflective) layer corresponding to the squamous epithelium, a highly scattering (hyper-reflective) layer corresponding to the lamina propria, a weakly scattering layer corresponding to the muscularis mucosae (difficult to recognize), a moderately scattering layer corresponding to the submucosa, and a weakly scattering, deep layer corresponding to muscularis propria.

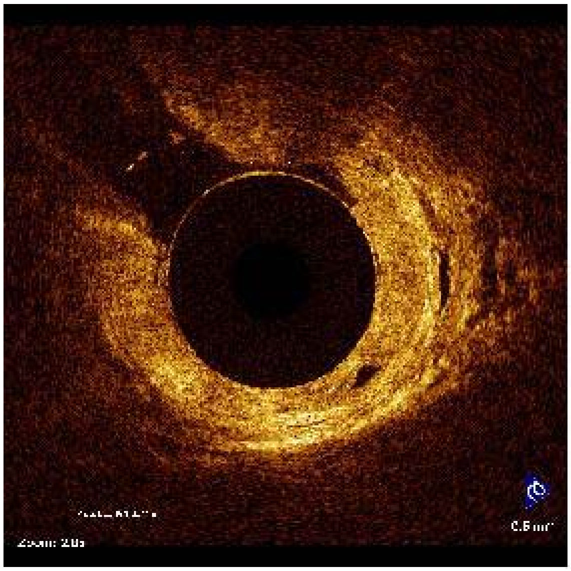

**FIGURE 2.** OCT imaging of the normal esophageal wall: a horizontal, layered tissue architecture appears clearly recognizable.

In normal conditions, the OCT image of the esophageal wall consists of a multiple-layer structure characterized by a superficial, weakly scattering (hyporeflective) layer corresponding to the squamous epithelium, a highly scattering (hyper-reflective) layer corresponding to the lamina propria, a weakly scattering layer corresponding to the muscularis mucosae, a moderately scattering layer corresponding to the submucosa, and a weakly scattering, deep layer corresponding to the muscularis propria (Fig. 1). The latter layer is not even recognizable *in vivo*, depending on the depth of penetration of the OCT probe used. Submucosal gland and vessels have been also identified[7,16]. In a recent *ex vivo* study, the muscularis mucosae was distinctly visualized during OCT investigation by using a Ti:Sapphire laser as light source[17]. Overall, the normal esophageal wall architecture shows a clearly recognizable horizontal, layered structure at OCT imaging (Fig. 2).

Barrett's epithelium is characterized by the presence of specialized intestinal metaplasia within the esophageal mucosa and has been the object of intense OCT research. The hallmark histologic feature of specialized intestinal metaplasia is the presence of goblet cells. Identification of Barrett's epithelium is of clinical relevance since the lesion requires endoscopic follow-up, being a recognized precancerous condition. In clinical practice, Barrett's epithelium is generally identified by performing multiple biopsies within the areas of gastric metaplasia, either in a random manner or after a previous vital staining with methylene blue.

OCT images of Barrett's epithelium show distinct features that enable the differentiation of intestinal metaplasia from other tissues. Although the intersubject variability of OCT imaging of normal squamous epithelium and gastric mucosa appears to be low, the OCT imaging of Barrett's epithelium demonstrated a greater variability in the published studies. OCT features predictive for the presence of intestinal metaplasia reported by Poneros at al. are (a) the absence of the layered structure of the normal squamous epithelium and the presence of the vertical crypt-and-pit morphology of normal gastric mucosa; (b) a disorganized architecture with inhomogeneous back-scattering of the signal and an irregular mucosal surface; (c) the presence of submucosal glands characterized as pockets of low reflectance below the epithelial surface at the OCT imaging[8,18] (Fig. 3). When these OCT criteria were applied to images acquired prospectively, the criteria were found to be 97% sensitive and 92% specific for specialized intestinal metaplasia, with a PPV (positive predictive value) of 84%. However, the presence of the crypt-and-pit architecture may render difficult the ability to discriminate between intestinal metaplasia and normal or inflamed gastric mucosa[9,19].

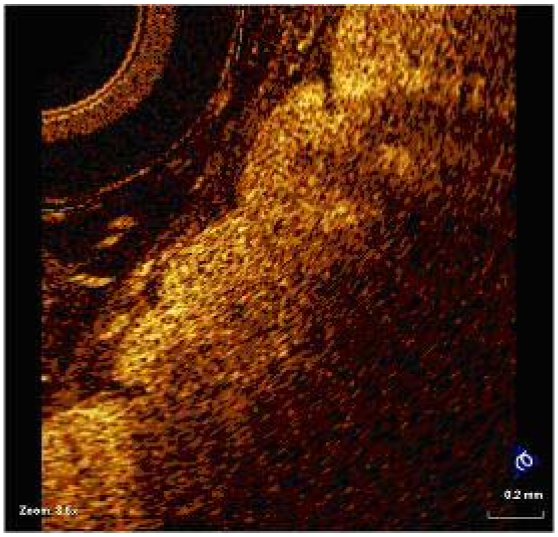

**FIGURE 3.** Barrett's esophagus. OCT features predictive for the presence of intestinal metaplasia are (a) the absence of the layered structure of the normal squamous epithelium and the presence of the vertical crypt-and-pit morphology of normal gastric mucosa; (b) a disorganized architecture with inhomogeneous back-scattering of the signal and an irregular mucosal surface; (c) the presence of submucosal glands characterized by a markedly hyporeflective tissue below the epithelial surface.

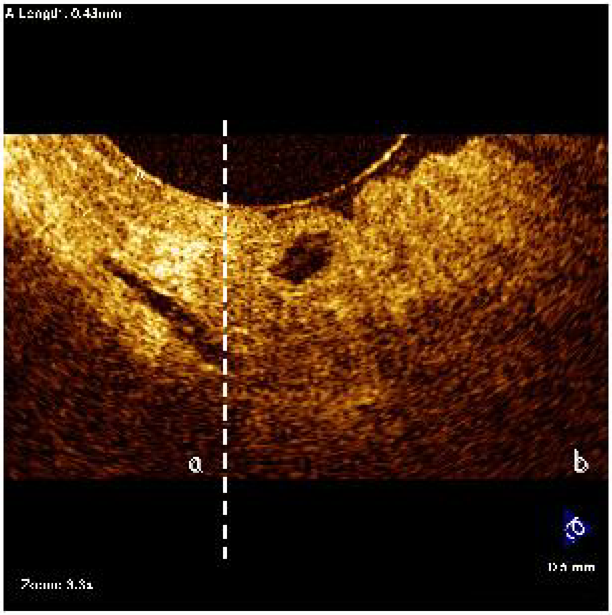

**FIGURE 4.** OCT imaging of normal squamous and Barrett's epithelium. At OCT imaging, normal esophageal wall shows a longitudinal, multiple-layer architecture determined by the squamous epithelium, lamina propria, muscularis mucosae, and submucosa (a). Barrett's epithelium shows a vertical crypt-and-pit architecture determine by the glandular structure (b).

In our experience, the OCT-magnified pattern of intestinal metaplasia tissue was mainly characterized by the presence of a larger superficial layer showing a markedly hyporeflective and inhomogeneous signal, depending on the presence of goblet cells, poorly differentiated from the hyper-reflective layer corresponding to the lamina propria. In general, the multiple-layer structure of the esophageal wall was maintained, although poorly defined.

Fig. 4 shows and compares OCT imaging in normal esophageal and Barrett's epithelium.

The diagnosis of dysplasia within the Barrett's epithelium is of crucial importance in the management of the disease. Unfortunately, up to now, attempts to identify OCT patterns characteristic for dysplasia, mainly the high-grade type, have been substantially disappointing. The increased nuclear-to-cytoplasmic ratio occurring in dysplasia may alter the light reflection characteristics, giving a more inhomogeneous back-scattering of the signal (Fig. 5). Because the degree of reflectivity depends on nuclear size, a markedly inhomogenous and hyporeflective back-scattering of the signal should indicate the presence of high-grade dysplasia (Fig. 6). Moreover, it is possible that by quantitating the OCT signal as a function of depth, OCT would be able to characterize high-grade dysplasia within intestinal metaplasia tissue. Poneros et al., by using two parameters of tissue reflectivity as an indicator of dysplasia, retrospectively diagnosed high-grade dysplasia with a 100% sensitivity and 85% specificity[18]. Such an accurate analysis of the degree of signal reflectivity requires avoidance of areas with incorrect artifact signal properties; this may be obtained by the identification of a precisely defined area with homogeneous signal reflectance, an adequate catheter-tissue contact, a reduction of motion artifacts (Fig. 7). Recently, the morphologic appearance of the OCT image, rather than a quantitative analysis of the OCT signal in the image, was used for the diagnosis and grading of dysplasia. For this purpose, an endoscope fitted with an Endoscopic Mucosal Resection (EMR) standard cap was used to stabilize the mucosal surface and negate movement from esophageal peristalsis and transmitted cardiac and respiratory motion. In this study, sensitivity, specificity, PPV, negative predictive value (NPV), and diagnostic accuracy for dysplasia were, respectively, 68, 82, 53, 89, and 78%[20]. In a more recent study, Evans et al. elaborated an OCT image scoring system of dysplasia ("dysplasia index") based on histopathologic features to validate OCT criteria for differentiating intramucosal carcinoma (IMC) and high-grade dysplasia (HGD) from low-grade dysplasia (LGD) in Barrett's esophagus. A total of 177 biopsy-correlated images were analyzed. By using the proposed "dysplasia index", the sensitivity and specificity for diagnosing IMC/HGD were 83 and 75%, respectively[21].

However, with the currently available OCT devices, the recognition of dysplasia within intestinal metaplasia and mainly the differentiation between LGD and HGD still appears difficult.

On the other hand, since ultrasound endoscopy cannot reliably detect the presence of dysplasia in Barrett's esophagus and differentiate between the low- and high-grade form, OCT, given its superior resolution in the investigation of the mucosal and submucosal layers, could become a powerful adjunct to standard endoscopy in detection and surveillance of Barrett's esophagus and HGD at an early stage[22].

OCT features characteristic for adenocarcinoma arising from Barrett's epithelium are the lack of the regular esophageal wall–layered morphology and a markedly heterogeneous back-reflectance of the signal[7,23] (Fig. 8). These features permit clear identification of the lesion and differentiation between the neoplastic and non-neoplastic tissue in advanced disease.

Fig. 9 shows and compares OCT findings of normal esophageal mucosa, Barrett's epithelium, dysplasia, and adenocarcinoma.

Studies are needed to evaluate whether OCT can identify and stage the lesion at an early stage. Since the penetration depth of OCT does not exceed 1–2 mm, the technique could be useful in staging superficial cancers that are difficult to stage accurately with ultrasound endoscopy (Figs. 10 and 11).

## Stomach

OCT images of the gastric mucosa are characterized by less contrast, depending on the crypt-and-pit architecture of the glandular epithelium[2,8,12]. Four layers can be identified from the surface: the glandular epithelium, muscularis mucosae, submucosa with blood vessels, and muscularis propria[9].

Inflammation, as occurs in gastritis, has been reported to produce greater back-scattering of the signal and a more pronounced crypt-and-pit pattern architecture, compared with normal tissue[18].

Gastric mucosa and esophageal squamous epithelium are easily recognizable at OCT imaging because of the different OCT pattern, characterized by the presence of the vertical crypt-and-pit architecure in the stomach and the horizontal, layer structure of squamous epithelium (Fig. 12). On the other hand, the differentiation between gastric and Barrett's epithelium appears more difficult.

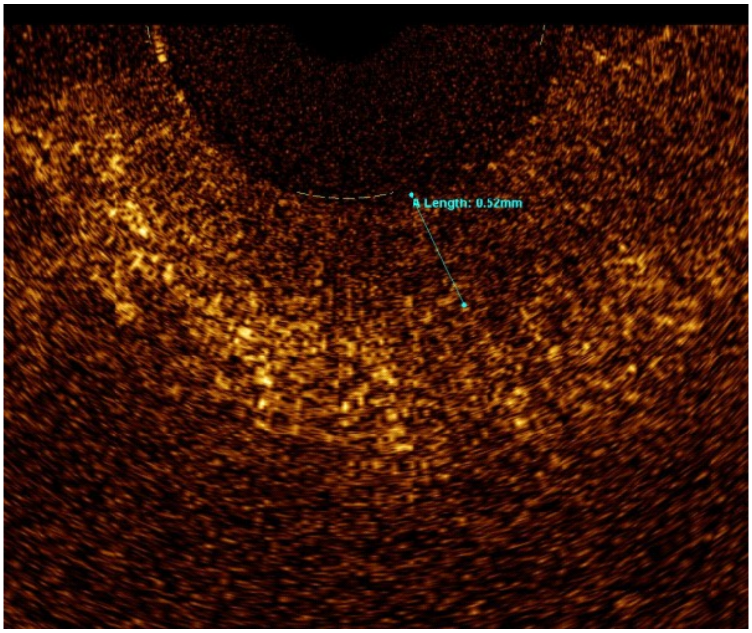

**OCT**

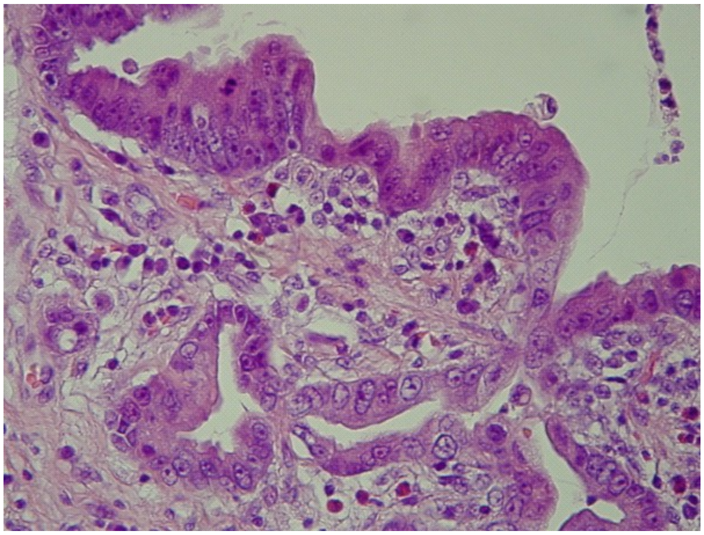

**Histology**

**FIGURE 5.** Magnified OCT imaging of dysplasia, compared with histology. The increased nuclear-to-cytoplasmic ratio occurring in dysplasia may alter the light reflection characteristics, giving a more inhomogeneous back-scattering of the signal. Layer surface between superficial epithelium and lamina propria appears poorly recognizable as the layered structure.

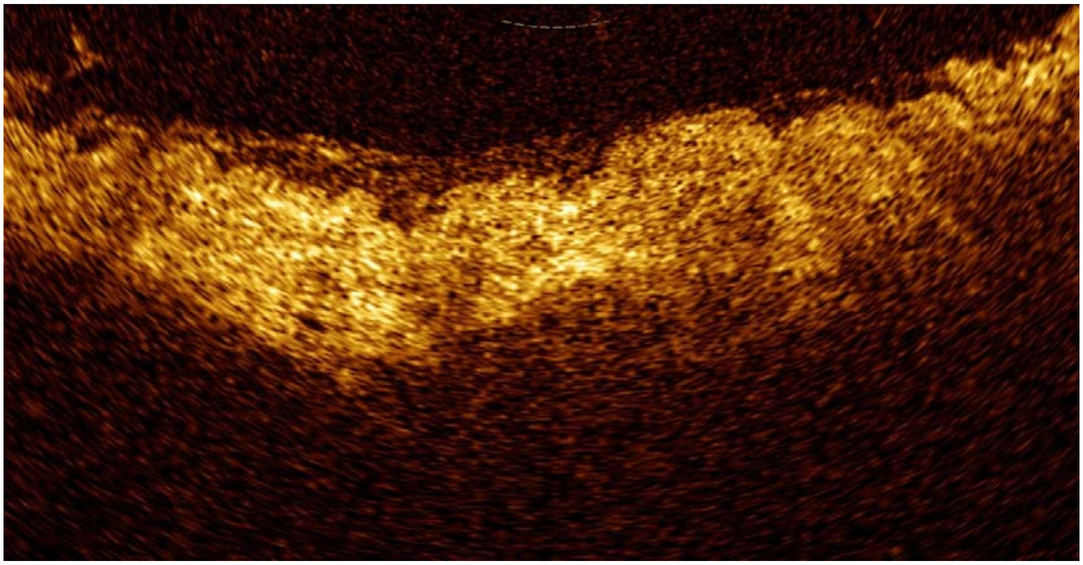

**FIGURE 6.** OCT image showing high-grade dysplasia. Because the degree of reflectivity depends on nuclear size, a markedly inhomogenous and hyporeflective back-scattering of the signal should indicate the presence of high-grade dysplasia.

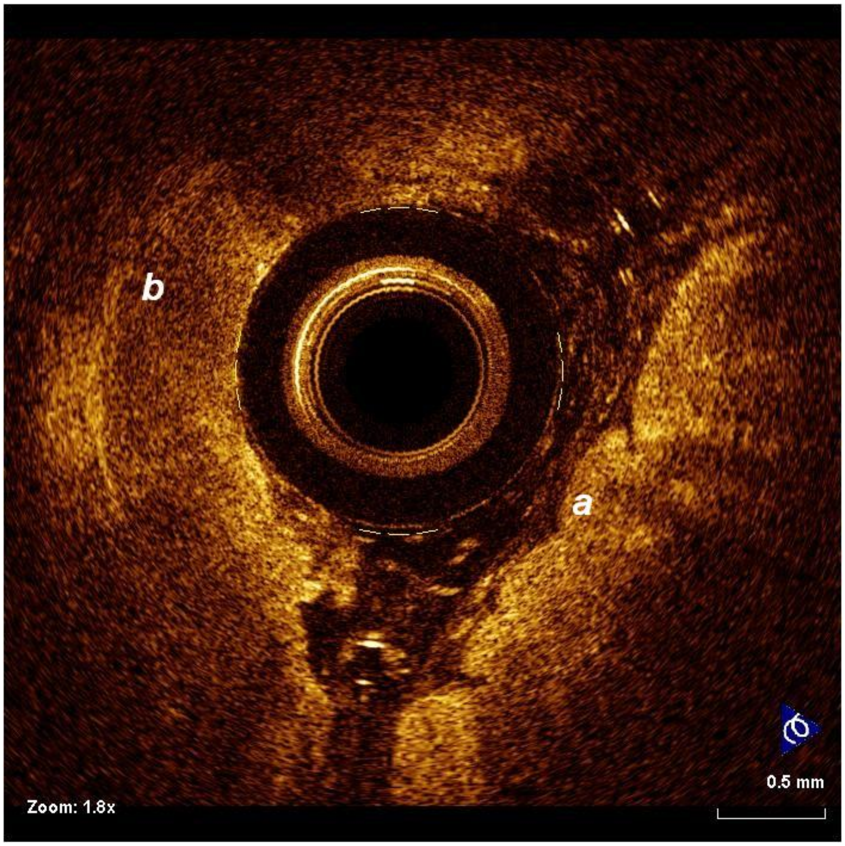

**FIGURE 7.** OCT imaging of Barrett's epithelium (a). An excessive pressure of the probe against the esophageal wall (b) may render difficult the evaluation of the epithelial structure.

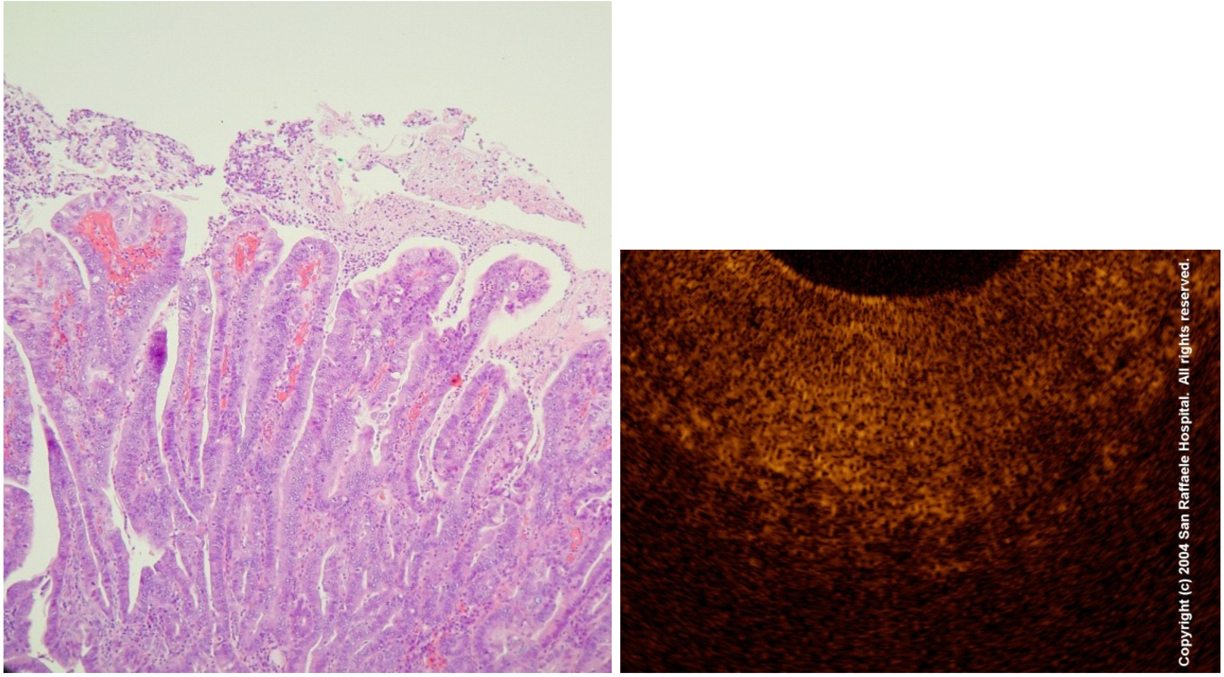

**FIGURE 8.** Magnified OCT imaging of adenocarcinoma compared with histology. Characteristic OCT features are the lack of the regular esophageal wall–layered morphology and a markedly heterogeneous back-reflectance of the signal.

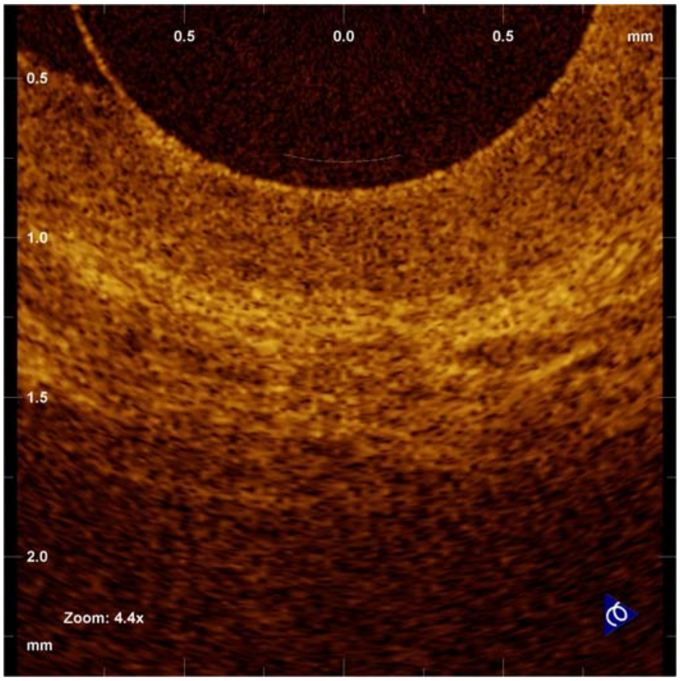

**A**

**FIGURE 9.** Comparison of OCT findings of normal esophageal mucosa (a), Barrett's epithelium (b), dysplasia (c), and adenocarcinoma (d).

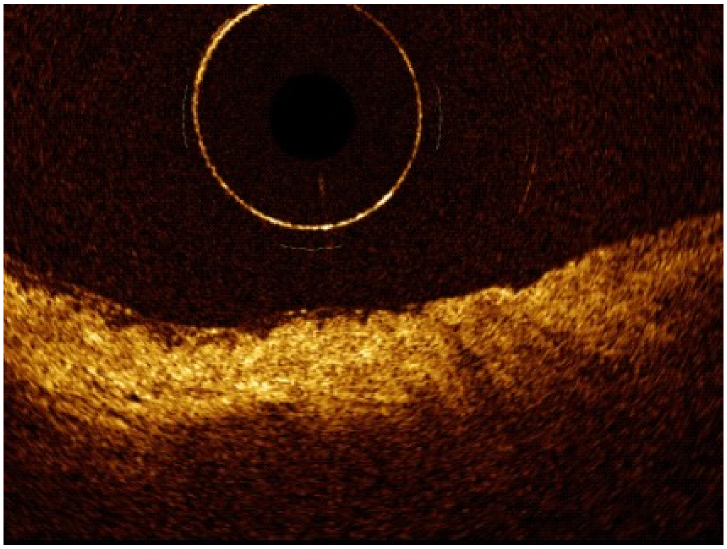

**FIGURE 9B**

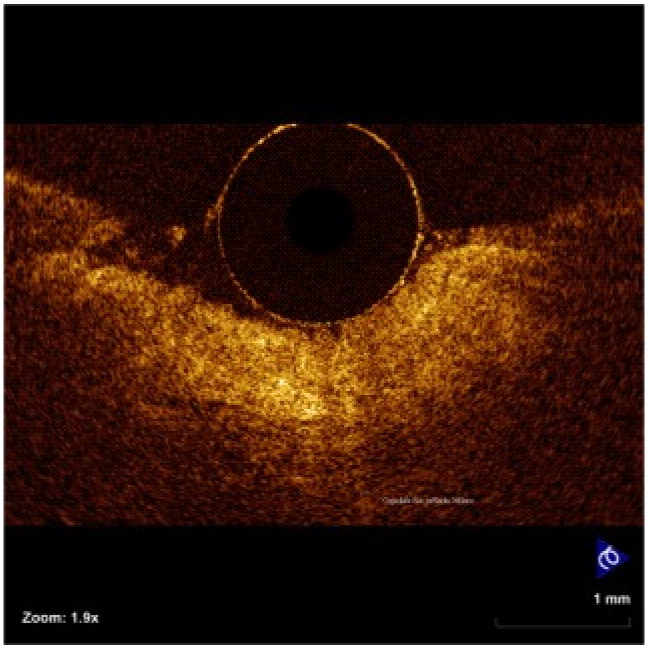

**FIGURE 9C**

## Duodenum, Small Intestine, and Colon

In the duodenum and small intestine, OCT clearly recognizes the mucosa and submucosa with the vascular structure[5,16]. OCT identified intestinal villous morphology and the degree of atrophy with 100% agreement compared to histology in a study by Hsiung et al., who analyzed OCT images *ex vivo* on fresh surgical specimens from the small intestine compared with histology[24]. In celiac disease, we found OCT able to recognize, with a very good concordance with histology, the normal villous pattern including their morphology and height, and the mild and severe villous atrophy[25] (Fig. 13). Unfortunately, current OCT devices are unable to recognize the cellular density that is a cornerstone in the diagnosis of celiac disease, so

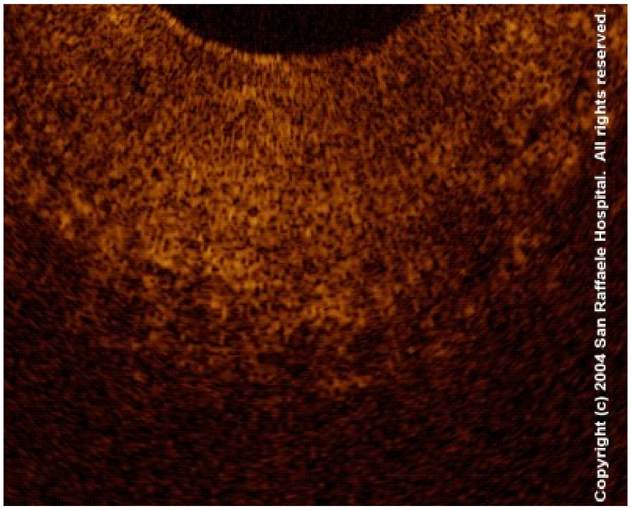

**FIGURE 9D**

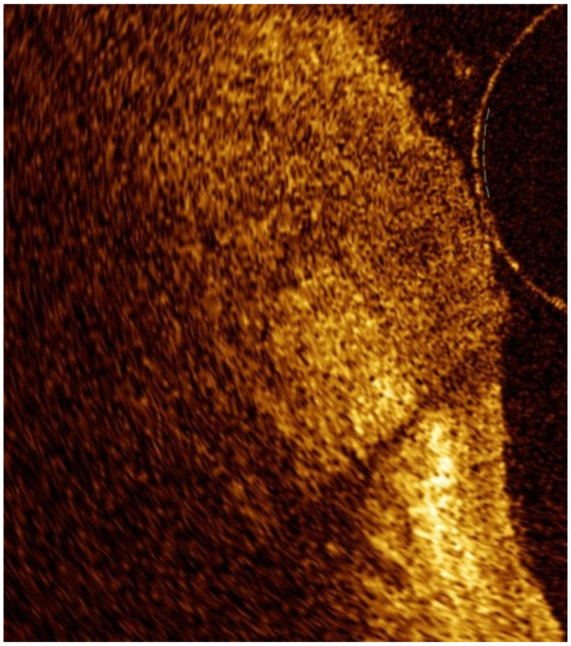

**FIGURE 10.** Magnified OCT imaging of early-stage adenocarcinoma arising within Barrett's epithelium. The lack of the regular esophageal wall–layered morphology and a markedly heterogeneous back-reflectance of the signal characterize the neoplastic lesion that is confined within the epithelium.

celiac patients who have normal villous morphology (Marsh I and II patients) cannot be identified. The ability of OCT imaging to recognize the villous pattern and its alterations could be used to identify celiac disease in real time during standard upper GI endoscopy, in patients undergoing endoscopy for conditions often related to a misdiagnosed celiac disease, such as iron deficiency anemia, osteoporosis, diabetes mellitus, or autoimmune disorders, and select dyspeptic patients who need biopsies for detecting the disease.

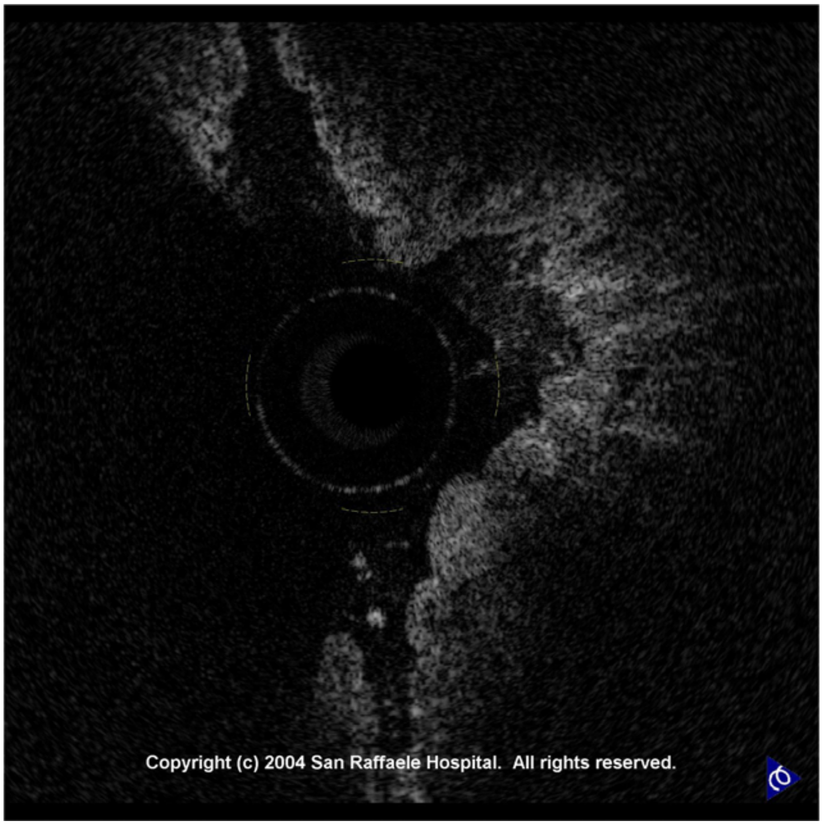

**FIGURE 11.** OCT imaging of early-stage adenocarcinoma arising within Barrett's epithelium. The lesion, clearly hyporeflective and inhomogeneous, invades the lamina propria and penetrates into the submucosal layer.

In the colon, the mucosa and submucosa can also be seen with strong correlation with histology. The mucosa appears as a hyper-reflective layer, submucosa as a hyporeflective layer with horizontal striations, and the OCT appearance is related to its composition, which could be of assistance in the diagnosis of chronic inflammatory conditions involving the submucosa. In the presence of dysplasia, the detection of a lower boundary of the mucosa may help in identifying the extension of dysplastic changes. Dynamic application of pressure of the OCT probe on the tissue reveals compressibility of both mucosa and submucosa that may be another criteria for identifying chronic inflammation and fibrosis[26]. The detection of transmural inflammation serves to distinguish patients with Crohn's disease from those with ulcerative colitis[27]. The detection of dysplasia may help in the follow-up of long-standing chronic inflammatory diseases[28].

Colonic polyps have also been investigated by means of OCT scanning that permitted differentiation between hyperplastic and adenomatous polyps[29].

## CLINICAL APPLICATION OF OCT IN THE PANCREATICO-BILIARY DUCTAL SYSTEM

The possibility to introduce the OCT probe into a standard transparent catheter for cannulation during an ERCP procedure permits investigation of the epithelial layers of the pancreato-biliary ductal system and sphincter of Oddi (Fig. 14).

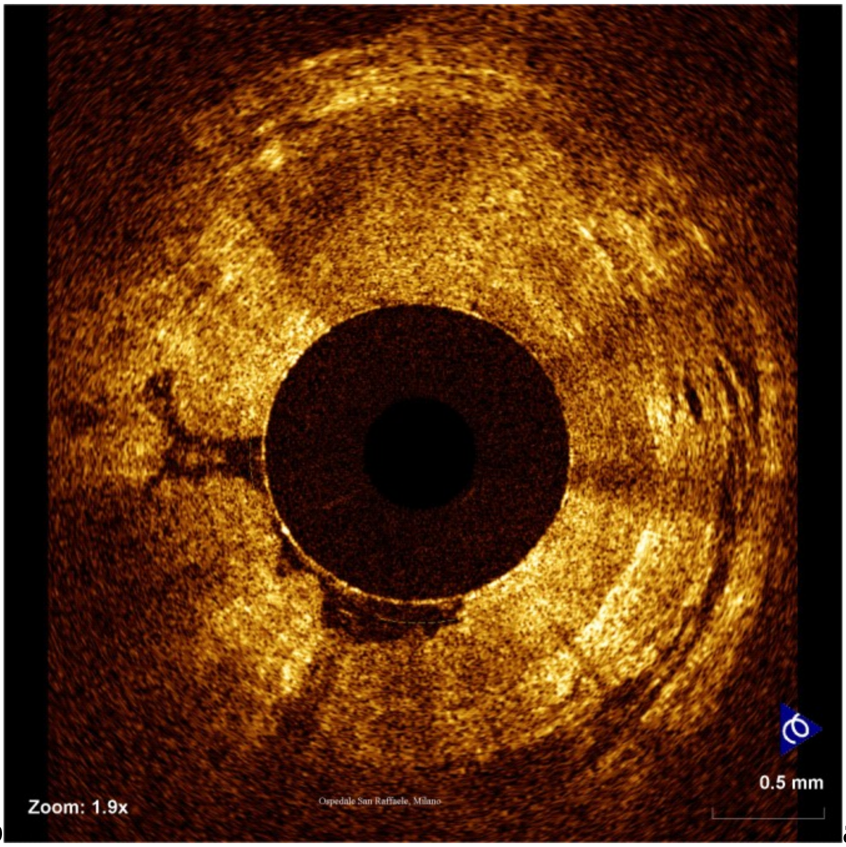

b a

**FIGURE 12.** OCT pattern of squamo-columnar junction. The horizontal, layered architecture of the esophageal wall (a) appears clearly distinguishable from the vertical crypt-and-pit architecture of the gastric wall (b).

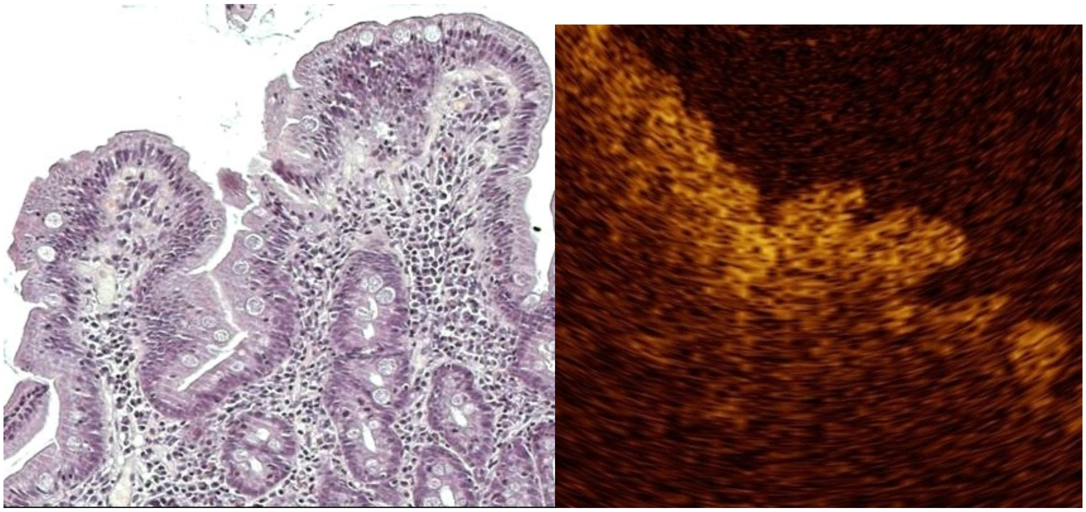

**FIGURE 13A.** Histological and OCT patterns of normal villous morphology (pattern 1).

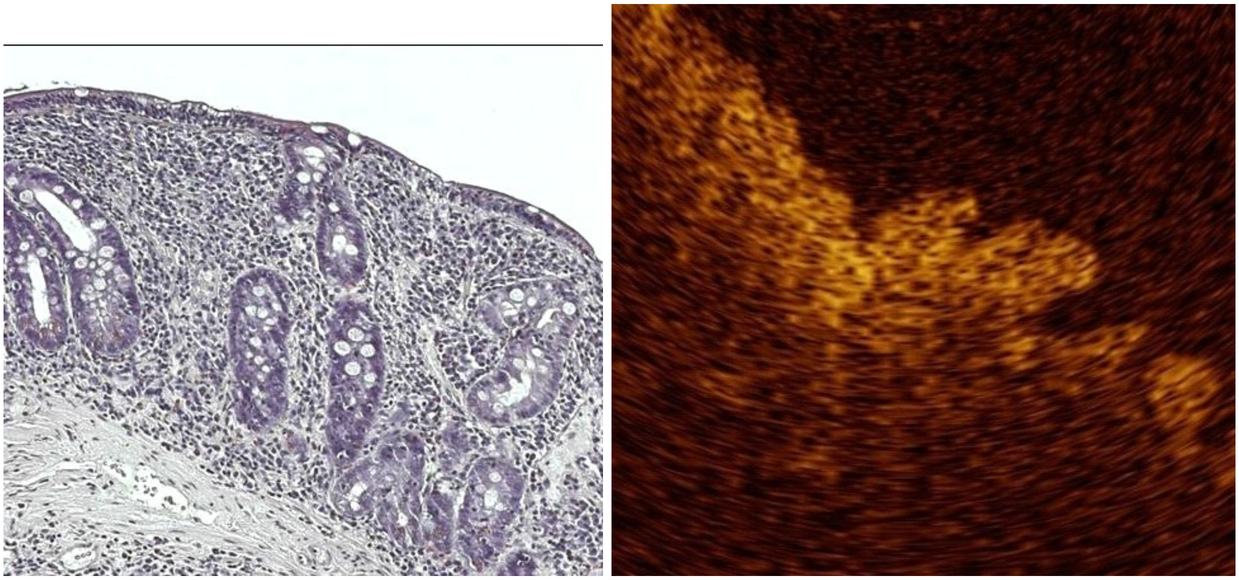

**FIGURE 13B.** Histological and OCT patterns of celiac duodenum with mild villous atrophy (pattern 3 a).

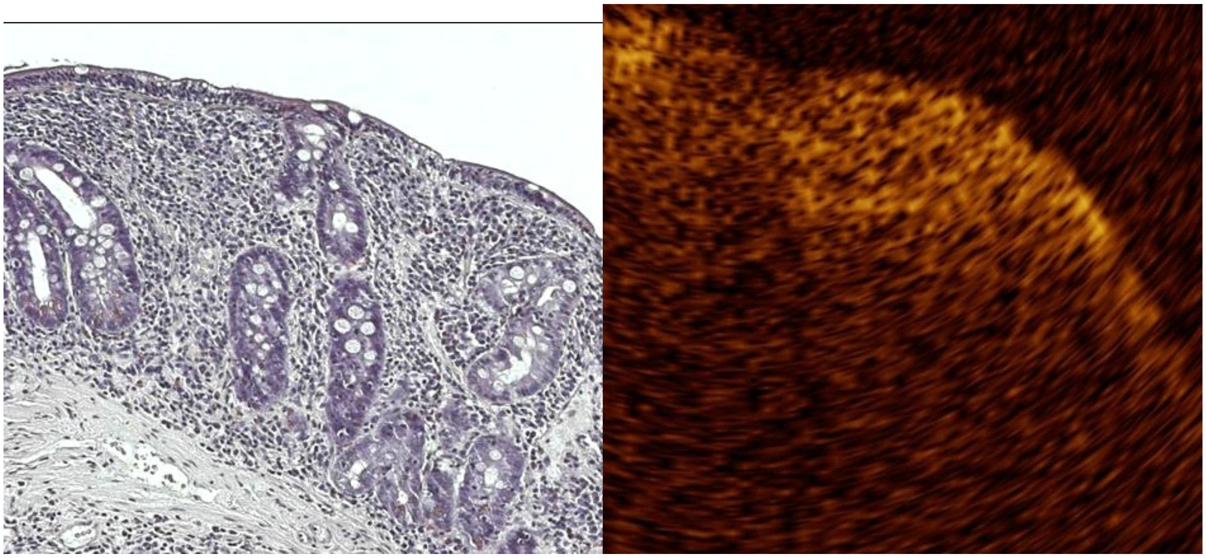

**FIGURE 13C.** Histological and OCT patterns of celiac duodenum with marked villous atrophy (pattern 3 b).

From a clinical point of view, OCT imaging of the pancreatic and biliary ductal system could improve the diagnostic accuracy for ductal epithelial changes and the differential diagnosis between neoplastic and non-neoplastic lesions since, in several conditions, X-ray morphology obtained by ERCP and other imaging techniques may be nondiagnostic and the sensitivity of intraductal brush cytology during ERCP procedures is highly variable.

## Normal Pancreatico-Biliary Ductal System

To date, visualization of the epithelium of the main pancreatic duct has been obtained mainly postmortem[30] and *ex vivo* in humans[6,31,32], while *in vivo*, it comes from one study in animals[33] and,

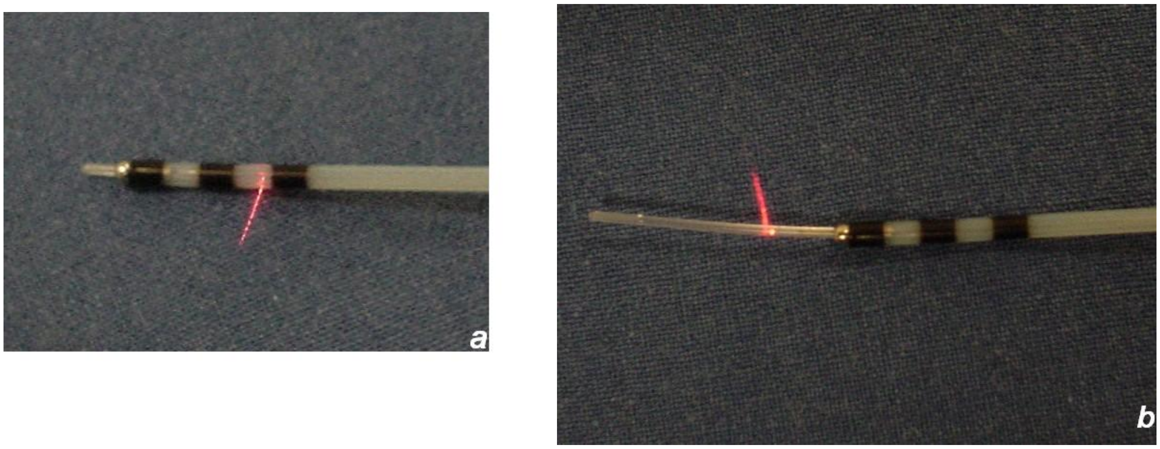

**FIGURE 14.** OCT probe inside (a) and outside (b) the ERCP catheter.

only recently, in humans by our group[34]. The normal biliary ductal system has been investigated in humans, *ex vivo* in a study[31] and *in vivo* in two ERCP-based studies[35,36].

In a recent study by our group[31], OCT imaging of the main pancreatic duct, common bile duct, and sphincter of Oddi normal structure has been shown to be able to provide features that were similar to those observed in the corresponding histological specimens in 80% of sections; the agreement between OCT and histology in the definition of normal wall was good (81.8%). OCT images identified three differentiated layers up to a depth of about 1 mm. From the surface of the duct, it was possible to recognize an inner hyporeflective layer corresponding to the single layer of epithelial cells close to the lumen; an intermediate, homogeneous, hyper-reflective layer corresponding to the fibromuscular layer surrounding the epithelium; and an outer, less-definite, hyporeflective layer corresponding to the smooth muscular structure within a connective tissue in the common bile duct and at the level of the sphincter of Oddi, and connective-acinar structure in the main pancreatic duct (Figs. 15, 16, and 17).

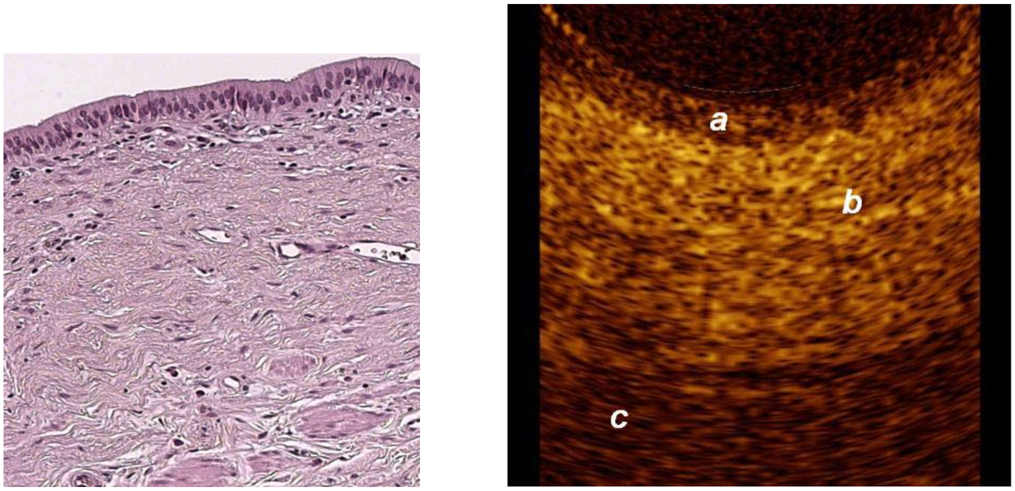

**FIGURE 15.** Magnification of an OCT image from the normal main pancreatic duct wall, compared with histology. From the surface of the duct, up to a depth of 1 mm, the following layers are recognizable: (a) the single layer of epithelial cells, approximately 0.04–0.08 mm thick, visible as a superficial, hyporeflective band; (b) the connective-fibro-muscular layer surrounding the epithelium, visible as a hyper-reflective layer approximately 0.36–0.56 mm thick; (c) the connective and acinar structure close to the ductal wall epithelium, visible as a hyporeflective layer.

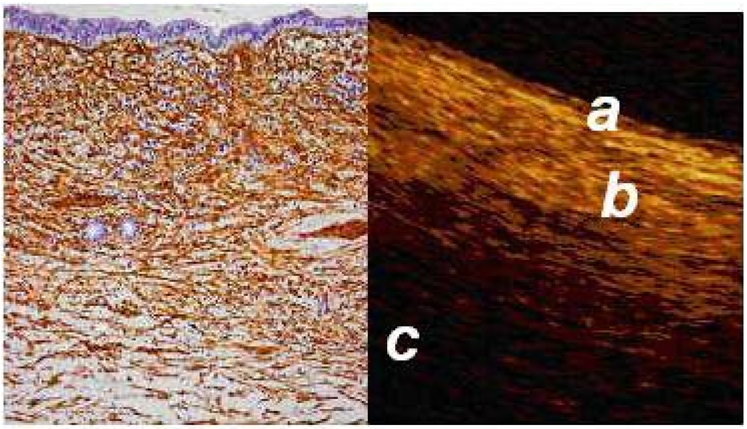

**FIGURE 16.** Magnification of an OCT image from the normal common bile duct wall, compared with histology. From the surface of the duct, up to a depth of 1 mm, the following layers are recognizable: (a) the single layer of epithelial cells, approximately 0.04–0.06 mm thick, visible as a superficial, hyporeflective band; (b) the connective-muscular layer surrounding the epithelium, visible as a hyper-reflective layer approximately 0.34–0.48 mm thick; (c) the connective layer visible as a hyporeflective layer with longitudinal relatively hyper-reflective strips (smooth muscle fibers).

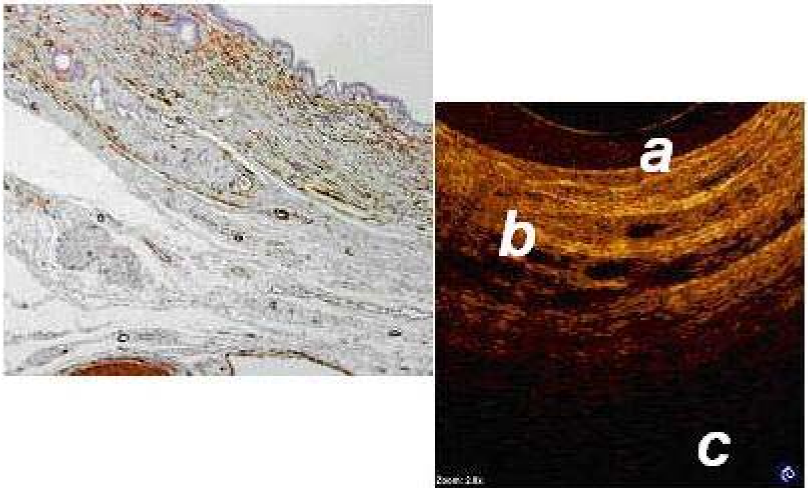

**FIGURE 17.** Magnification of an OCT image from the normal sphincter of Oddi wall, compared with histology. From the surface of the duct, up to a depth of 1 mm, the following layers are recognizable: (a) the single layer of epithelial cells, approximately 0.04–0.08 mm thick, visible as a superficial, hyporeflective band; (b) the connective-muscular layer surrounding the epithelium, visible as a hyper-reflective layer approximately 0.23–0.37 mm thick; (c) the connective layer visible as a hyporeflective layer with longitudinal relatively hyper-reflective strips (smooth muscle fibers). Within the intermediate and outer layer, vessels are also recognizable, visualized as nonreflecting areas surrounded by a hyporeflective endothelium. Margins between the intermediate and outer layer are poorly recognizable due to the irregular distribution of connective and muscular structure.

The inner, hyporeflective layer showed a mean thickness of 0.05 mm (range: 0.04–0.08 mm) and a homogeneous back-scattering of the signal in all the imaged sites; thickness, surface regularity, and reflectance degree of this layer did not substantially differ in the common bile duct, main pancreatic duct, and sphincter of Oddi. The intermediate layer showed a mean thickness of 0.41 mm (range: 0.34–0.48 mm) in the common bile duct, 0.42 mm (range: 0.36–0.56 mm) in the main pancreatic duct, and 0.29 mm (range: 0.23–0.37 mm) in the sphincter of Oddi. The layer thickness was substantially similar in the ducts, while it appeared reduced by 25% at the level of the sphincter of Oddi. The layer appeared hyper-reflective when compared with the inner and outer layers, and the reflectance degree did not change in all the imaged sites. Within the context of the intermediate layer, tiny, multiple, nonreflective areas were recognizable in the main pancreatic duct and at the level of sphincter of Oddi. The outer layer appeared recognizable until a depth of about 1 mm from the lumen and was hyporeflective in all the imaged sites. Multiple, hyper-reflective, longitudinal strips were recognizable at the level of the common bile duct and sphincter of Oddi. These longitudinal strips were more pronounced and hyper-reflective in the sphincter of Oddi, so at this level, the layer appeared less hyporeflective than in the common bile duct.

The three different layers showed a linear, regular surface and each layer had a homogeneous back-scattered signal in every frame; however, the differentiation between the intermediate and outer layer appeared more difficult than between the inner and intermediate layer. The thickness of the inner and intermediate layers measured by OCT was similar to those measured by histology; the muscular and connective-acinar structure was visible until the working depth of penetration into the tissue of the near-focus probe (about 1 mm).

Smooth muscle structure appeared at OCT scanning as hyper-reflective, longitudinal strips within a context of hyporeflective tissue and were particularly recognizable at the level of sphincter of Oddi. Veins, arteries, and secondary pancreatic ducts were also identifiable by OCT, characterized by hypo- or nonreflective, well-delimited areas (Fig. 18).

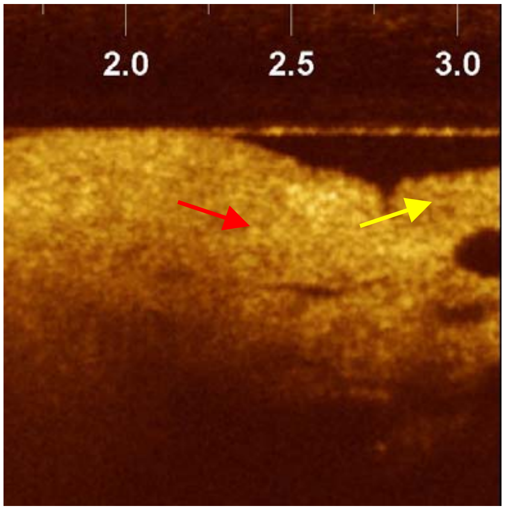

**FIGURE 18.** OCT image from the normal main pancreatic duct wall visualized by linear array OCT: a portion of an accessory duct is visible close to the pancreatic duct (yellow arrow) as a nonreflective, well-delimited area, with larger diameter than observed for blood vessels (red arrow).

The images acquired in this study provided information on tissue architectural morphology that could have only previously been obtained with conventional biopsy. These results suggest that OCT could become a powerful imaging technology, enabling high-resolution diagnostic images to be obtained from the pancreato-biliary system during a diagnostic ERCP procedure.

Although OCT images were obtained from surgical specimens, the early evaluation (within 1 h from pancreatic resection) of ductal OCT-imaged morphology was not likely to have been influenced by artifacts induced by autolytic tissue degradation occurring in autopsy specimens, as reported in previous studies[30,36]. The data obtained *ex vivo* from this study can therefore be considered useful for *in vivo* evaluation too.

## Pathological Pancreatico-Biliary Ductal System

The pathological pancreatic ductal system has been investigated by our group in humans in two *ex vivo* studies[7,32] performed on multiple surgical pancreatic specimens obtained from patients with pancreatic head adenocarcinoma.

In chronic inflammatory changes involving the main pancreatic duct, OCT still showed a conserved, three-layer architecture. However, the inner, hyporeflective layer appeared slightly larger than normal (0.07–0.24 mm) and the intermediate layer appeared more hyper-reflective than in normal tissue; this is probably because of the dense mononuclear cell infiltrate. The back-scattered signal was heterogeneous with marked hypo- or hyper-reflectance in some sections.

The agreement between OCT and histology in the definition of Main Pancreatic Duct (MPD) chronic inflammatory changes was poor (27.7%).

The OCT pattern in the presence of dysplasia of the main pancreatic duct epithelium was characterized by an inner layer markedly thickened (0.49 mm), strongly hyporeflective, and heterogeneous; this OCT finding is probably due to the initial structural disorganization (increased mitosis and altered nucleus/cytoplasm ratio). The surface between the inner and intermediate layers appeared irregular. As in chronic inflammatory tissue, dysplasia too gave strong hyper-reflectance of the intermediate layer, particularly in the part closest to the inner layer. The outer layer did not differ from other nonmalignant conditions and appeared homogeneously hyporeflective.

However, in chronic pancreatitis and dysplasia, OCT and histology were concordant only in 62% of cases. The K statistic used to assess agreement between the two procedures was equal to 0.059 for non-neoplastic MPD wall appearance.

Overall, normal wall structure and chronic inflammatory or low-grade dysplastic changes cannot be distinguished in 38% of the sections because the architecture of the layers and surface light reflection did not show a characteristic OCT pattern.

In all sections with histologically proven adenocarcinoma, OCT showed a totally subverted MPD wall architecture. The three layers of the ductal wall and their linear, regular surface, normally giving a homogeneous back-scattered signal, were not recognizable. The margins between the connective-fibro-muscular layer and acinar tissue were unidentifiable. The back-scattering of the signal appeared strongly heterogeneous, with minute, multiple, nonreflective areas in the disorganized pancreatic microstructure. In 100% of sections with adenocarcinoma, OCT and histology were concordant. A totally subverted wall architecture was also observed by OCT in the presence of neoplastic tissue within the common bile duct[36].

Fig. 19 shows magnified OCT images from sections of main pancreatic duct with normal tissue, chronic pancreatitis, low-grade dysplasia, and adenocarcinoma.

Studies *in vivo* were performed in animals[33] and humans[34]. We evaluated the diagnostic accuracy of OCT for the diagnosis of carcinoma, during ERCP, in a series of patients with MPD strictures of unknown etiology. In this study, the accuracy of OCT for detection of neoplastic tissue was 100%, compared with 66.7% for intraductal brush cytology. The study showed that OCT is feasible during an ERCP procedure and was superior to brush cytology in distinguishing non-neoplasstic from neoplastic lesions[34] (Fig. 20).

In conclusion, OCT appears a promising technique for real-time, high-resolution, cross-sectional imaging of the mucosa of the GI tract and pancreatico-biliary ductal system during routine endoscopic procedures. It has been shown to be a highly sensitive and specific means of identifying neoplastic tissue and preneoplastic lesions, and distinguishing between non-neoplastic and neoplastic tissue. To date, the technique has been documented to be particularly useful in the investigation of esophageal mucosa and pancreatico-biliary ductal system. Given its superior resolution compared with other imaging modalities,

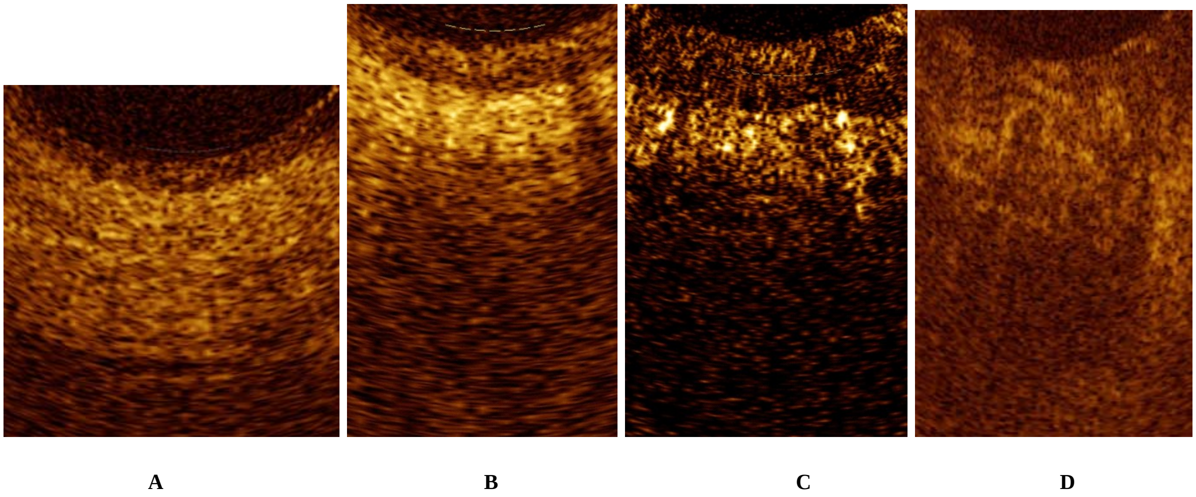

**FIGURE 19.** Magnified OCT images from sections with either normal (a), tumor-associated chronic inflammatory (b), low-grade dysplasia (c), and adenocarcinoma (d) tissue.

such as EUS, OCT has great potential as a powerful adjunct to standard endoscopy in identification, and mainly in the surveillance of Barrett's epithelium in order to detect high-grade dysplasia and adenocarcinoma an early stage. The technique recognizes with high definition the mucosa, muscularis mucosae, and submucosa, and could therefore identify cases in which mucosectomy becomes a curative procedure. In the pancreatico-biliary ductal system, OCT can be used to discriminate between non-neoplastic and neoplastic tissue when strictures of unknown etiology are identified during an ERCP procedure, being its diagnostic accuracy higher than reported for intraductal brush cytology.

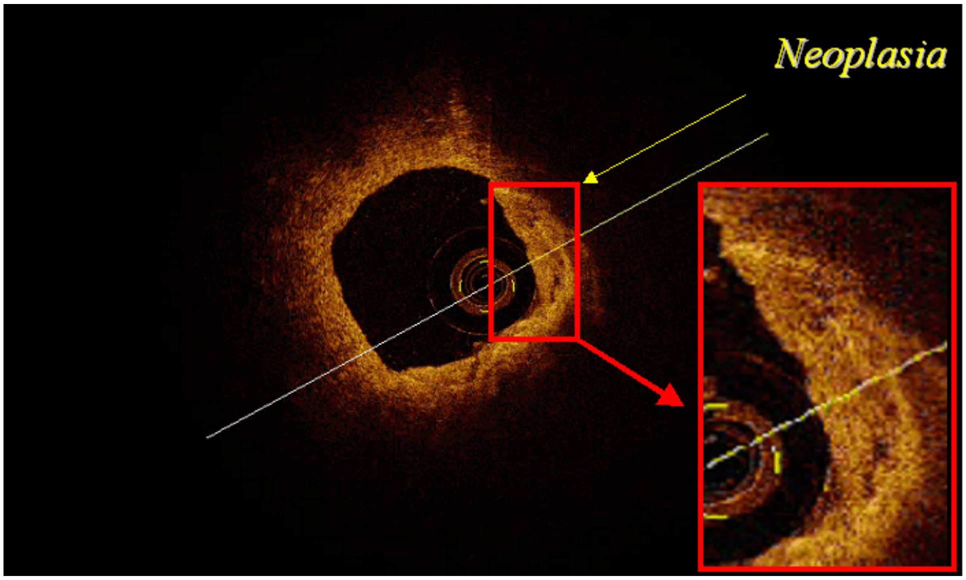

**FIGURE 20.** Adenocarcinoma of the common bile duct at early stage, detected by OCT during ERCP. Within a normal tissue, OCT recognizes a delimited area showing a subverted wall architecture, with loss of the ductal layers and unidentifiable connective and muscular layer. The three layers and their linear and regular surface, normally giving a homogeneous back-scattered signal, are not recognizable. This OCT image has a heterogeneous back-scattered signal with minute, multiple, nonreflective areas (necrotic areas) in the disorganized CBD microstructure.
